# *MDR1* gene polymorphisms and HIV therapeutic response in South Indian population

**DOI:** 10.1186/1471-2334-14-S3-O17

**Published:** 2014-05-27

**Authors:** Shruthi Lingaiah, Anita Shet, Arun S Shet, Ujjwal Neogi

**Affiliations:** 1 Hematology Research Unit, Division of Molecular Medicine, St John’s Research Institute, Bangalore, India; 2 Division of Public health and Infection Disease Epidemiology, St John’s Research Institute, Bangalore, India

## Background

Multi drug resistance (*MDR1*) gene polymorphisms can alter the drug transport function of p-glycoprotein and are often associated with resistance to antiretroviral therapy (ART) in HIV-1 patients. In this study, we investigated the association of single nucleotide polymorphisms in *MDR1* gene (exon-21 and exon-26) with responsiveness to ART in HIV-1 patients.

## Methods

Eighty HIV-1 infected drug naive patients who had atleast one viral load <150 copies/mL within six months of therapy and 21 healthy subjects were included. The HIV patients were categorized into viral responders (n=40) and non responders, two consecutive viral load ≥400 copies/mL after six months of ART (n=40).The genotype analysis of G2677T (GG/GT/TT in exon-21) and C3435T (CC/CT/TT in exon-26) was performed using PCR RFLP. Statistical analysis was carried out using chi-square test in SPSSver16.

## Results

The proportion of CC, CT and TT-genotype among the healthy individuals was found to be 0.09, 0.52 and 0.38 whereas GT and TT-genotype was 0.61 and 0.38 respectively (No GG-genotype). Among HIV-1 patients, the distribution of CC, CT and TT-genotype between responders and non responders was (0.15 vs 0.07), (0.37 vs 0.50) and (0.50 vs 0.42) and GG, GT and TT was (0.12 vs 0.05), (0.30 vs 0.55) and (0.57 vs 0.40) respectively. Only the GT-genotype was significantly higher in non responders compared to responders (*p*=0.023).

## Conclusion

The GT-genotype appears to predict poorer response to ART compared to other genotypes. Further studies could define more clearly the prognostic value of *MDR1* gene polymorphisms in determining therapeutic response to ART.

